# Peritoneal immune microenvironment of endometriosis: Role and therapeutic perspectives

**DOI:** 10.3389/fimmu.2023.1134663

**Published:** 2023-02-14

**Authors:** Siman Chen, Yukai Liu, Zhiqi Zhong, Chunyan Wei, Yuyin Liu, Xiaoyong Zhu

**Affiliations:** ^1^ Laboratory for Reproductive Immunology, Hospital of Obstetrics and Gynecology, Fudan University, Shanghai, China; ^2^ Xinglin College, Nantong University, Nantong, Jiangsu, China; ^3^ Department of Gynecology and Obstetrics, The Third Affiliated Hospital of Guangzhou Medical University, Guangzhou, China; ^4^ Key Laboratory of Reproduction Regulation of NPFPC, SIPPR, IRD, Fudan University, Shanghai, China; ^5^ Shanghai Key Laboratory of Female Reproductive Endocrine Related Diseases, Fudan University, Shanghai, China

**Keywords:** endometriosis, immune microenvironment, macrophage, natural killer (NK) cells, immunotherapy

## Abstract

Endometriosis, an estrogen-dependent chronic inflammatory disease characterized by the growth of endometrium-like tissues outside the uterine cavity, affects 10% of reproductive-age women. Although the pathogenesis of endometriosis is uncertain, it is widely accepted that retrograde menstruation results in ectopic endometrial tissue implantation. Given that not all women with retrograde menstruation develop endometriosis, immune factors have been hypothesized to affect the pathogenesis of endometriosis. In this review, we demonstrate that the peritoneal immune microenvironment, including innate immunity and adaptive immunity, plays a central role in the pathogenesis of endometriosis. Current evidence supports the fact that immune cells, such as macrophages, natural killer (NK) cells, dendritic cells (DCs), neutrophils, T cells, and B cells, as well as cytokines and inflammatory mediators, contribute to the vascularization and fibrogenesis of endometriotic lesions, accelerating the implantation and development of ectopic endometrial lesions. Endocrine system dysfunction influences the immune microenvironment through overexpressed estrogen and progesterone resistance. In light of the limitations of hormonal therapy, we describe the prospects for potential diagnostic biomarkers and nonhormonal therapy based on the regulation of the immune microenvironment. Further studies are warranted to explore the available diagnostic biomarkers and immunological therapeutic strategies for endometriosis.

## 1 Introduction

Endometriosis, a chronic inflammatory disease, is defined as the growth of endometrium-like tissues outside the uterine cavity, at locations such as the peritoneum, ovary, and cervix. The typical clinical symptoms of endometriosis are progressive dysmenorrhea, chronic pelvic pain, deep dyspareunia, and infertility, all of which affect the patient’s quality of life (1). It is estimated that 10% of reproductive-age women are affected by endometriosis (2). Although the etiology and pathogenesis of endometriosis remain indistinct, the retrograde menstruation theory proposed by Sampson in 1921 is widely accepted. Besides, there are other hypotheses of the pathophysiology of endometriosis, including coelomic metaplasia and vascular and lymphatic metastasis (3). However, none of them were able to adequately explain all pathological types of endometriosis. Additionally, it is generally accepted that immune, endocrine, genetic, and environmental factors are also involved in the pathogenesis of endometriosis (4).

Retrograde menstruation refers to the reflux of menstrual debris containing endometrial cells through the fallopian tubes into the peritoneal cavity, where they are implanted in the peritoneum and pelvic organs (5). Some studies suggest that not all women with retrograde menstruation develop endometriosis and those with innate or adaptive immune disorders are more likely to have it (6). After menstrual debris flows into the abdominal cavity, the innate and adaptive immune systems are activated and try to clear the fragments. The immune cells infiltrate and begin to repair the peritoneal cavity. As time passes, persistent endometrial debris may lead to immune system overload, contributing to immune dysfunction (7). Immune microenvironment dysfunction improves the adhesion, proliferation, and invasion of the endometrial cells, contributing to the pathogenesis of endometriosis. Moreover, cytokines in the peritoneal fluid (PF) can directly stimulate the proliferation and invasion of endometrial cells and neovascularization, accelerating the progression of endometriotic lesions (8).

The endocrine system plays a central role in the pathogenesis of endometriosis. Estrogen overexpression and progesterone resistance lead to immune system dysfunction and affect the immune microenvironment of the peritoneal cavity. In addition to surgical treatment, the current medical treatment for endometriosis is hormonal therapy. In light of the side effects and relatively poor efficacy of hormonal therapy, new nonhormonal treatments such as immunotherapy are urgently required. Targeted immunotherapy aimed at regulating the immune microenvironment for cancer has made great progress in recent years (9). Immunotherapeutic targets are comprised of specific genetic mutations, clusters of differentiation molecules, and immune checkpoint molecules on immune cells, such as CD25, CTLA-4, and programmed death-1 (PD-1) (10–12). Adoptive cell immunotherapy, including the chimeric antigen receptor (CAR)-T cell and the engineered T-cell receptor (TCR)-T cell, has been developed to boost the anticancer immune microenvironment (13). Consequently, immunotherapy for endometriosis has great potential to provide more specific and effective therapeutic alternatives with fewer side effects.

In this article, we discuss the role of the peritoneal immune microenvironment in the pathogenesis of endometriosis and provide future perspectives on nonhormonal treatment based on the regulation of the immune microenvironment.

## 2 The immune microenvironment in the peritoneal cavity of endometriosis

**Figure 1:**
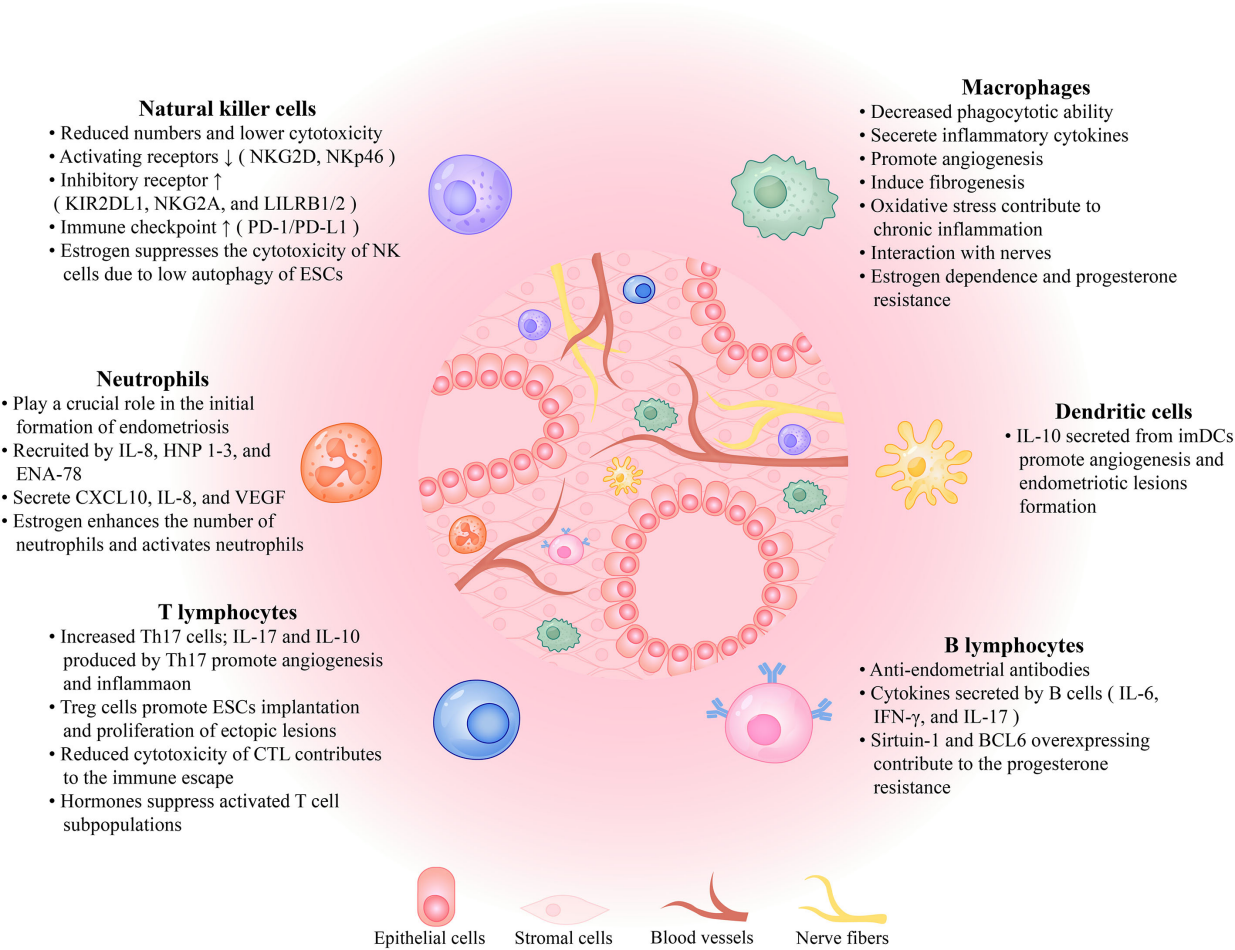
The immune microenvironment in the peritoneal cavity of the patient with endometriosis. Endometriotic lesions are comprised of epithelial cells, stromal cells, blood vessels, and nerve fibers that interact with immune cells, such as macrophages, natural killer cells, dendritic cells, neutrophils, B lymphocytes, and T lymphocytes, as well as a variety of cytokines, all of which contribute to the formation of the immune microenvironment. The role each component plays in the growth and development of endometriotic lesions is described here. NKG2D, NK Group 2, Member D; LILRB1/2, leukocyte immunoglobulin-like receptor B1/2; PD-1/PD-L1, programmed cell death protein 1/programmed cell death ligand 1; ESCs, endometrial stromal cells; IL, interleukin; HNP 1-3, human neutrophil peptides 1, 2, and 3; ENA-78, epithelial neutrophil-activating protein-78; VEGF, vascular endothelial growth factor; Th17 cells, T helper 17 cells; CTL, cytotoxic T lymphocytes; imDCs, immature dendritic cells; IFN-γ, interferon-γ; BCL6, B cell lymphoma 6.

Peritoneal immune microenvironment dysfunction has been proposed as a critical facilitator of endometriotic lesion growth after retrograde menstruation (**Figure 1**). The reflux of menstrual fragments induces innate and adaptive immune responses. Therefore, the immune cells infiltrate and try to repair the peritoneum. Subsequently, the cytokines released by immune cells contribute to the chronic inflammatory response and enable endometriotic lesions to grow and implant. In the initial stage of endometriosis, the pro-inflammatory environment takes the dominant position; however, during the advanced phase, the environment tends toward immune tolerance (14).

### 2.1 The innate immune system of endometriosis

Many studies have demonstrated that the innate immune system plays a central role in the pathogenesis of endometriosis, including the immune cells, chemokines, and other components. There are macrophages, natural killer (NK) cells, dendritic cells (DCs), and neutrophils in the innate immune system of the peritoneal immune microenvironment.

#### 2.1.1 Monocytes & Macrophages

**Figure 2:**
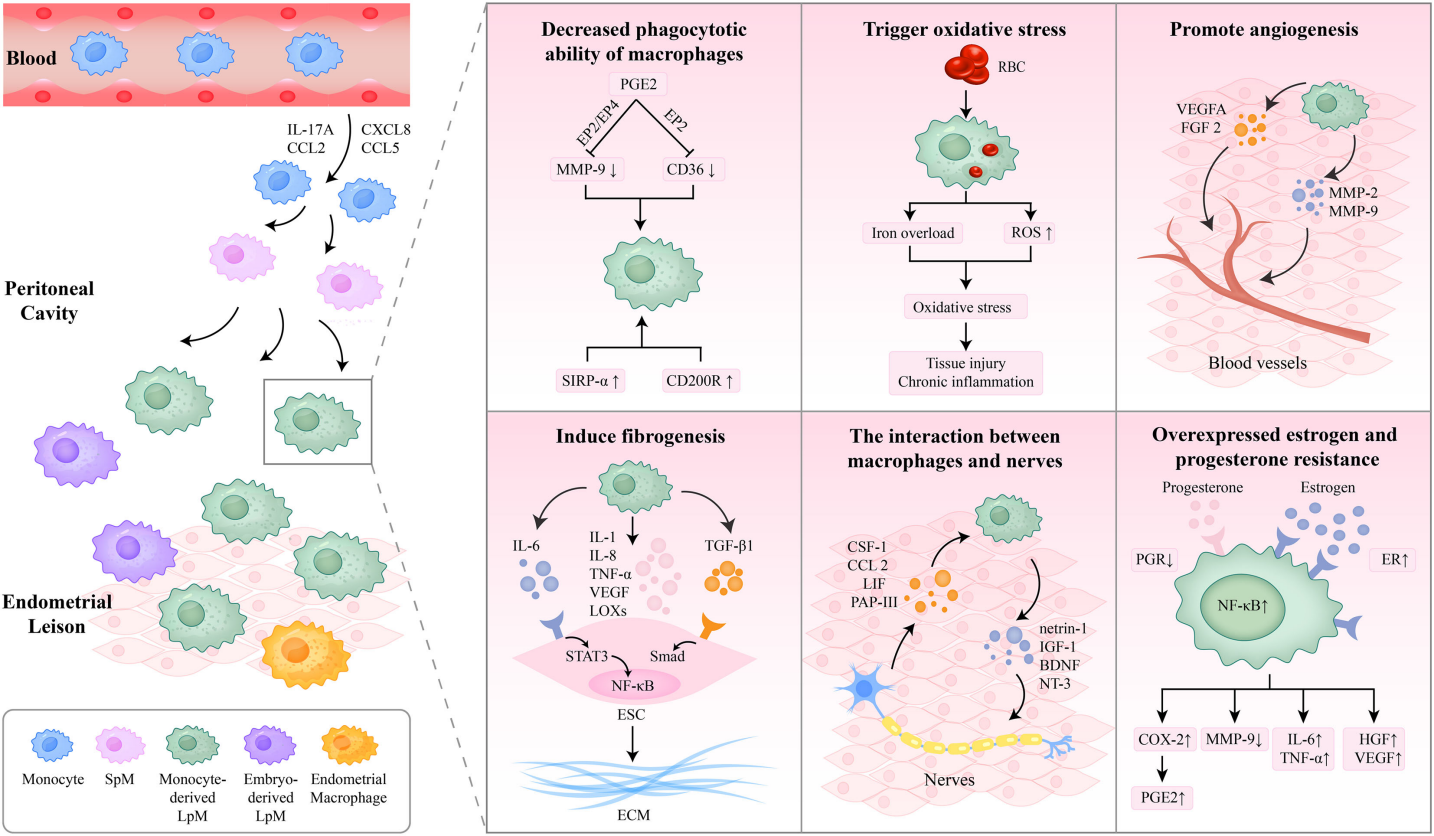
The role macrophages play in the pathogenesis of endometriosis. The macrophages in the endometriotic lesions are composed of embryo-derived LpM and SpM, eutopic endometrial macrophages, and monocyte-derived LpM. Monocyte-derived macrophages were recruited into the peritoneal cavity and replenished the LpM pool. The decreased phagocytotic capacity of macrophages contributes to ESC survival and endometriotic tissue growth. Oxidative stress in macrophages triggers oxidative damage, tissue injury, and chronic inflammation. Macrophages promote angiogenesis and fibrosis through a variety of cytokines. Furthermore, the interaction between macrophages and nerves promotes the development of endometriosis. Additionally, overexpressed estrogen and progesterone resistance influence the function of macrophages. IL, interleukin; SpM, small peritoneal macrophages; LpM, large peritoneal macrophages; PGE2, prostaglandin E2; MMP, matrix metalloproteinase; SIRP-α, signal regulatory protein-α; RBC, red blood cell; ROS, reactive oxygen species; VEGF, vascular endothelial growth factor; FGF, fibroblast growth factor; TNF-α, tumor necrosis factor-α; LOXs, lysyl oxidases; TGF, transforming growth factor; NF-κB, nuclear factor kappa B; ESC, endometrial stromal cell; ECM, extracellular matrix; CSF, colony-stimulating factor; LIF, leukemia inhibitory factor; PAP-III, pancreatitis-associated protein III; IGF-1, insulin-like growth factor-1; BDNF, brain-derived neurotrophic factor; NT-3, neurotrophin-3; PGR, progesterone receptor; ER, estrogen receptor; COX-2, cyclo-oxygenase 2; HGF, hepatocyte growth factor.

Previous studies have established that the number of macrophages is increased in the PF and endometrial lesions of patients with endometriosis *via* double immunofluorescence staining and flow cytometry (15, 16). In healthy women without endometriosis, macrophages have phagocytotic properties that enable them to eliminate menstrual debris and maintain homeostasis. Conversely, many studies have supported the hypothesis that macrophages play a critical role in endometriotic lesion growth owing to their decreased phagocytotic ability, promoting angiogenesis and fibrogenesis, chronic inflammation induced by oxidative stress, interactions with nerves, estrogen dependence, and progesterone resistance (**Figure 2**). Additionally, many studies found increased levels of cytokines secreted by macrophages in PF samples collected from patients with endometriosis, such as interleukin (IL)-1β, IL-6, IL-8, tumor necrosis factor (TNF)-α, and transforming growth factor (TGF)-β, which resulted in a peritoneal inflammatory environment (17).

##### 2.1.1.1 Origins, recruitment, and phenotypes of macrophages

The macrophages in the peritoneal cavities of patients with endometriosis originate from eutopic endometrial tissues, resident peritoneal macrophages, and blood monocytes derived from bone marrow progenitors. Their different origins decide their functions (18, 19). Hogg C et al. conducted experiments on mouse models by depleting different populations differently and observed that depleting macrophages from the eutopic endometrium reduced the number of endometriosis lesions while inhibiting monocytes recruitment increased the number of endometriosis lesions and reduced the total number of peritoneal macrophages. Macrophages from the eutopic endometrium, which are regarded as “pro-endometriosis,” promote the development of endometriosis lesions. Resident peritoneal macrophages are composed of embryo-derived large peritoneal macrophages (LpM) and small peritoneal macrophages (SpM) derived from monocytes and DCs. They demonstrated that monocyte-derived macrophages were recruited into the peritoneal cavity and replenished the LpM pool, proposed as “anti-endometriosis,” protecting the peritoneal cavity from the establishment of endometriosis lesions (19).

Following the reflux of menstrual debris, monocytes are recruited into the peritoneal cavity by a variety of chemokines, where they clear cell debris and heme-iron (20). Miller et al. investigated whether IL-17A could induce macrophage recruitment and M2 polarization and promote endometriotic lesion growth through enhanced vascularization using *in vitro* and *in vivo* models (21). In addition, some scholars hold the view that the endometriosis-induced expression of monocyte chemotactic protein-1 (MCP-1, also known as chemokine ligand 2, CCL2) contributes to paracrine and autocrine activation and macrophage recruitment (22). Other chemokines such as CXCL8 (IL-8) and CCL5 (RANTES) also play a role in macrophage recruitment (23).

Depending on the activation pathway, macrophages are classified as “classically activated” (M1) or “alternatively activated” (M2) macrophages. It is widely acknowledged that M2 macrophages promote the development of endometriosis but not M1 macrophages (24, 25). Bacci suggested that M2 macrophages promote the vascularization and growth of ectopic endometrial lesions in a mouse model, while M1 macrophages inhibit the growth of these lesions (24). Wang et al. harbored the idea that fractalkine (FKN), which is secreted by eutopic endometrial stromal cells (ESCs), will enhance the production of IL-10 and inhibit the production of IL-12, inducing the M2 polarization of macrophages that is beneficial to the proliferation and invasiveness of ESCs (25). Additionally, Nie et al. found that the upregulation of Smad2/Smad3 occurs in macrophages exposed to eutopic and ectopic endometrial homogenates of women with endometriosis, supporting the hypothesis of M1 to M2 macrophage polarization *via* the Smad2/Smad3 pathway (26). On the contrary, Takebayashi and Vallvé-Juanico held the opposite view that M1 macrophages are the more abundant macrophage population in the endometrium of endometriosis patients (27, 28). Additionally, some experts argued that macrophages should not be easily divided into two subsets since they interact to form complex and mixed phenotypes (29).

##### 2.1.1.2 Decreased phagocytotic ability of macrophages

Macrophages play an essential role in the development of endometrial lesions, contributing to ectopic cell survival and tissue vascularization (20). A majority of reports support the hypothesis that the macrophages in the PF have decreased phagocytotic ability (1, 7). Our previous study has demonstrated that elevated concentrations of heme (which is released by lysed erythrocytes) in the PF of patients with endometriosis impair the phagocytic ability of macrophages (30). The eutopic endometrium could reduce the phagocytic ability of peritoneal macrophages in women with endometriosis by increasing the expression of signal regulatory protein-α (SIRP-α) and suppressing the expression of CD36 (31). SIRP-α is a phagocytosis inhibitor, while CD36 is a scavenger receptor that aims to clear oxidized low-density lipoprotein and apoptotic neutrophils. Additionally, Chuang et al. indicated that prostaglandin E2 (PGE2) inhibited the expression of CD36 in peritoneal macrophages *via* the EP2 receptor-dependent signaling pathway, reducing their phagocytic ability (32). Wu et al. suggest that the suppression of matrix metalloproteinase (MMP)-9 by PGE2 *via* the EP2/EP4-dependent PKA pathway contributes to the decreased phagocytotic ability of peritoneal macrophages (33). Correspondingly, the expression of CD200 in ectopic endometrial tissues and the CD200 receptor (CD200R) in peritoneal macrophages of women with endometriosis are increased by estrogens, resulting in the decreased phagocytic capacity of macrophages (34).

##### 2.1.1.3 Oxidative stress based on macrophages results in chronic inflammation

Oxidative stress based on macrophages also plays an essential role in the pathogenesis of endometriosis. Macrophages phagocytose senescent erythrocytes, which are carried into the pelvic cavity by retrograde menstruation. The metabolism of hemoglobin and heme by heme oxygenase (HO) releases iron, which is either stored in the form of ferritin or hemosiderin in macrophages or binds to the iron transporter, transferrin (Tf), which transports it into the PF (35). The continuous delivery of iron to macrophages leads to iron overload and abnormal macrophage activation, inducing iron-mediated damage and oxidative stress (36, 37). Reactive oxygen species (ROS) produced by macrophages in oxidative stress play an essential role in the regulation of the transcriptional factor nuclear factor-kappa B (NF-kB), which has been implicated in the pathogenesis of endometriosis (38). Oxidative stress in the pelvic cavity triggers oxidative damage, tissue injury, and chronic inflammation, leading to the proliferation and fibrosis of ectopic endometrial lesions (39).

##### 2.1.1.4 Macrophages promote the angiogenesis of endometriotic lesions

Numerous studies have shown that macrophages can produce angiogenic growth factors, including vascular endothelial growth factor A (VEGFA) and fibroblast growth factor 2 (FGF 2), which play a role in vascularization, accompanied by hypoxic environments such as tumors and endometriosis (40). Macrophages also secrete proteolytic enzymes, such as MMP-2 and MMP-9, which cleave and remodel the extracellular matrix (ECM) to promote angiogenesis. Sekiguchi et al. treated WT endometriosis-affected mice with clophosome N, which deleted macrophages in the peritoneal cavity, and found that both endometrial tissue growth and angiogenesis were suppressed (41). A recent report suggests that CD206^+^ macrophages, which are classified as M2 macrophages, promote the formation of endometriotic-like lesions by inducing angiogenesis around them (42).

##### 2.1.1.5 Macrophages induce the fibrogenesis of endometriotic lesions

Macrophages play a crucial role in inducing fibrogenesis in endometriotic lesions. Duan J et al. conducted experiments on mice models and found that macrophage depletion after the induction of endometriosis significantly reduced lesional fibrotic content and lesion weight, while the adoptive transfer of M2a macrophages increased the extent of fibrosis in lesions (43). M2a macrophages activated by IL-4 can secrete TGF-β1 and activate ESCs *via* the TGF-β1/Smad3 signaling pathway, producing ECM products *in vitro* (44). *In vivo*, TGF-β is an important profibrotic cytokine that promotes the proliferation and matrix production of fibroblasts and the deposition of collagen *via* TGF-β/Smad signaling (45). Ono Y et al. demonstrated that enhanced levels of Sphingosine 1-phosphate (S1P) in the PF stimulate M2 macrophage polarization and secret IL-6 and cyclo-oxygenase (COX)-2, resulting in inflammation and fibrosis in lesions (46). Furthermore, macrophages produce mediators that are beneficial to fibrogenesis, such as TNF-α, IL-1, IL-6, IL-8, VEGF, and lysyl oxidases (LOXs) (47–49). IL-6/soluble IL-6 receptor (sIL-6R) signaling dysfunction leads to the persistent activation of STAT3 and NF-κB, which induces the collagen synthesis of ESCs, contributing to the fibrosis of endometriosis (50).

##### 2.1.1.6 The interaction between macrophages and nerves in endometriotic lesions

Recently, a series of studies have confirmed that the interaction between macrophages and nerves promotes the development of endometriosis. On the one hand, nerve fibers in ectopic endometrial lesions will secrete colony-stimulating factor (CSF)-1 and CCL2, which attract macrophages to the periphery of nerves (51) and regulate the polarization of macrophages toward the M2 phenotype (52). Furthermore, there are other factors secreted by nerves that attract macrophages, such as the leukemia inhibitory factor (LIF) and pancreatitis-associated protein III (PAP-III) (53, 54). Additionally, Sema3A is an axon-repulsive guidance factor for neurons, and Sema3A/NRP-1 signaling recruits macrophages to the hypoxic microenvironment, such as the peritoneal cavity of endometriosis, and polarize macrophages from the M1 phenotype toward the M2 phenotype (55). Therefore, it may be considered a potential medium in the interaction between macrophages and nerves. On the other hand, macrophages also play a crucial role in the growth of nerve fibers by secreting various modules. Greaves et al. incubated macrophages exposed to CSF-1 with E2 and observed increased concentrations of brain-derived neurotrophic factor (BDNF) and neurotrophin-3 (NT-3), which stimulate neurite outgrowth (51). Recent studies have illustrated that neurotrophic factors participate in endometriosis-associated pain, which is considered a type of neuropathic pain (56, 57). Ding et al. suggested that netrin-1, an axon guidance cue produced by macrophages, promotes neural regeneration after injury in endometriotic lesions, which may play an essential role in the pathogenesis of endometriosis-associated pain (56). Another report demonstrated that macrophage-derived insulin-like growth factor-1 (IGF-1) is a crucial neurotrophic and nerve-sensitizing factor involved in the pathogenesis of endometriosis-related pain (57). In general, interactions between macrophages and nerve fibers result in neuroinflammation and inflammation-induced pain in endometriosis (58).

##### 2.1.1.7 Estrogen dependence and progesterone resistance in macrophages

Endocrine system dysfunction influences the function of macrophages through overexpressed estrogen and progesterone resistance.

Overexpressed estrogen and the estrogen receptor (ER) on macrophages of the peritoneal cavity generate an abnormal immune microenvironment. The increased production of estrogen and decreased metabolism of 17β-estradiol are triggered by aromatase and 17β-hydroxysteroid dehydrogenase (17β-HSD), which are secreted by endometrial lesions (59). The overexpression of ER-β in ESCs increases the production of MCP-1, recruiting macrophages *via* NF-κB signaling (60). Recently, Liu Y et al. illustrated that estrogen affected the polarization of PF macrophages in endometriosis *via* NF-κB activation (61). E2 induces the COX-2 expression and increases levels of PGE2 in PF macrophages by the action *via* ER-β, which reduces the expression and enzymatic activity of MMP-9 and inhibits phagocytic activity (62). As mentioned previously, estrogen stimulates the expression of CD200R in PF macrophages of women with endometriosis and decreases their phagocytic capacity (34). Furthermore, the enhanced expression of ER-α and ER-β in macrophages of women with endometriosis is correlated with the production of inflammatory cytokines (63). Khan KN et al. observed the increased secretion of IL-6 and TNF-α in macrophages cultured with E2 and lipopolysaccharide (LPS) (64). Another study demonstrated that estrogen stimulated PF macrophages to produce hepatocyte growth factor (HGF) and VEGF by ER, and HGF has a synergistic effect with E2 in the pathogenesis of endometriosis (65). Although it is known that estrogen functions by binding to the ER on macrophages, the exact mechanisms involved remain unclear. Further studies are warranted to explore the specific signal pathways as therapeutic targets.

Progesterone resistance in the endometriotic lesions of women with endometriosis which results from decreased progesterone receptor (PGR) expression and dysregulation in PGR signaling mediators, has been widely accepted (66). Progesterone decreases the number of activated macrophages and the expression of pro-inflammatory cytokines, suppressing E2-dependent inflammatory responses (67). Maeda N et al. observed that the dienogest could increase the expression of human leucocyte antigen (HLA)-DR in macrophages and decrease the production of TNF-α in PF samples collected from women with endometriosis, resulting in the enhancement of the antigen-presenting ability of macrophages and the anti-inflammatory microenvironment, and reduced endometriotic tissues (68).

Shen HH et al. demonstrated that dysfunction of the hormones-autophagy-immunity axis plays an essential role in the pathogenesis and progression of endometriosis (69). During menstruation, autophagy serves as a self-digestion mechanism to maintain homeostasis. On account of the overexpressed estrogen and progesterone resistance, altered autophagy-related genes lead to decreased endometrial autophagy. Estrogen suppresses ESC autophagy *via* NF-κB signaling by the CXCL12/CXCR4 interaction. Interestingly, progesterone also stimulates autophagy *via* the CXCL12-CXCR4 axis. Furthermore, the hypoxia environment of the peritoneal cavity in patients with endometriosis activates the COX-2/PGE2 pathway, stimulating M2 macrophage differentiation and the development of endometriosis (69).

#### 2.1.2 NK cells

In endometriosis patients, the main population of natural killer (NK) cells in the peripheral circulation is that of CD16^+^/CD56^dim^ NK cells, which are characterized as highly cytotoxic, while the main population in the endometrium and PF is that of CD16^-^/CD56^bright^ NK cells, which produce high-level cytokines (70). Furthermore, some studies found that the percentages of CD56^+^ NK cells in PF samples collected from women with endometriosis were significantly decreased (71, 72). Nevertheless, other researchers argued that there are no differences in CD56^+^ NK cells in the PF between patients and individuals in the control groups (73, 74). Considering that the data was collected twenty years ago, further studies are warranted to figure out whether CD56^+^ NK cells count is decreased in the PF of women with endometriosis.

After the reflux of endometrial debris, NK cells reject the endometrial cells, playing a role in immune surveillance. Many previous studies have reported decreased numbers of killer activating receptors (KAR) and increased numbers of killer inhibitory receptors (KIR) reduced cytotoxicity of NK cells in the PF of endometriosis patients, resulting in immune escape (73, 75–79). Consequently, retrograde endometrial cells are prone to survive in the peritoneal cavity, resulting in the development of endometriosis. Guo et al. reported that platelet-derived TGF-β1 suppresses the expression of NK Group 2, Member D (NKG2D), a KAR on NK cells, contributing to the reduced cytotoxicity of NK cells in women with endometriosis, while the neutralization of TGF-β1 reverses the reduction (80). Similarly, Du et al. reported that platelets impair NK cell reactivity and function through multiple mechanisms in endometriosis by conducting experiments in which mice models with induced endometriosis experienced either platelet depletion, NK cell depletion, both, or none (81). They found reduced KAR NKG2D and NKp46 expression and increased KIR2DL1 expression in NK cells. The impaired NK cell cytotoxicity was due to reduced NK cell degranulation and interferon (IFN)-γ production. In addition, they performed co-incubation of target cells with platelets, showing that the expression of NKG2D ligands, MHC class I chain-related proteins A and B (MICA, MICB), was reduced. Moreover, increased MHC-I expression on target cells could protect them from NK cells by connecting them to inhibitory receptors (81). Many experts supported the hypothesis that increased shedding of MICA and MICB contributed to the inhibition of NK cell activation and the evasion of immuno-surveillance (82, 83). Higher expression of the inhibitory receptor, CD94/NKG2A, on NK cells in the PF of patients with endometriosis, by reaction with its ligand human leukocyte antigen (HLA)-E, resulted in the inhibition of the cytotoxic function of NK cells (84). What is more, leukocyte immunoglobulin-like receptor B1 (LILRB1) and LILRB2 on NK cells bind to their ligand HLA-G, contributing to the impaired cytotoxicity of NK cells and immune escape (85). Besides, the immune checkpoint, PD-1/PD-L1, is also increased in estrogen-regulated ectopic endometrial lesions, inhibiting the function of NK cells and contributing to immune dysfunction (86).

Previous studies have demonstrated that some chemokines in the PF of women with endometriosis reduced NK cell cytolytic activity. Increased IL-6 in the PF could downregulate the expression of Src homology region 2-containing protein tyrosine phosphatase-2 (SHP-2), suppressing cytolytic granule components, such as granzyme B and perforin, which results in the reduction of NK cell cytolytic activity (87). Yang et al. constructed the co-culture systems of NK cells, macrophages, and ESCs from patients with endometriosis and discovered that the expression of CD16, NKG2D, perforin, and IFN-γ was significantly downregulated, while the secretion of IL-1β, IL-10, and TGF-β was increased (88). It was concluded that the interaction between ESCs and macrophages impairs the cytotoxicity of NK cells in endometriosis by secreting IL-10 and TGF-β.

Furthermore, hormones could suppress the cytotoxic activity of NK cells on account of the low autophagy of ESCs and facilitate the proliferation of ectopic endometrial lesions. Mei J et al. demonstrated that estrogen inhibits the CXCL12/CXCR4-mediated autophagy of ESCs, contributing to their survival (89). Moreover, they also suggested that the estrogen-autophagy-STAT3-HCK-NK cell axis participates in the pathogenesis of endometriosis. The reduction in the expression of hematopoietic cellular kinase (HCK) *via* STAT3 signaling increase in the expression of IL-8 and IL-23A by ESCs contributes to the reduction of the cytotoxic activity of NK cells through the depression of microRNA MIR1185-1-3p, targeted gene PTGS2 (90). In a co-culture system of ESCs and NK cells, an increase in IL-15 secretion by ESCs decreased the concentration of Granzyme B, IFN-γ, and NKG2D in NK cells, contributing to the impairment of the cytotoxicity of NK cells and the immune escape of ectopic ESCs (91).

#### 2.1.3 Dendritic cells

Unlike the macrophages and NK cells, the role of dendritic cells (DCs) in the pathogenesis of endometriosis is not well-defined. Fainaru et al. suggested that DCs play an essential role in the promotion of angiogenesis and lesion formation in endometriosis after they are injected into the peritoneal cavity of endometriosis mouse models (92). Pencovich et al. demonstrated that endometriotic lesions were five times smaller after the depletion of DCs by diphtheria toxin injections administered in the control group (93). On the contrary, Stanic et al. demonstrated that depleting DCs increased lesion sizes (94). Given these controversial results, further research is required to determine whether DCs promote or inhibit ectopic endometrial lesion formation in mouse models.

It has been reported that the concentration of DCs in the peritoneal fluid or endometrial lesions of women with endometriosis does not differ significantly from that of women without endometriosis, whatever is identified by the HLA-DR^+^CD11c^+^CD123^+^ lineage (95) or specific surface antigens (BDCA1, BDCA2, and BDCA3) (96). On the other hand, Schulke et al. demonstrated that the density of endometrial CD83^+^ DCs, classified as mature DCs (mDCs), was significantly decreased in women with endometriosis compared with those in the control group. In contrast, the density of CD1a^+^ DCs, which are classified as immature DCs (imDCs), was significantly increased (97). As a result, DCs are hypothesized to inhibit the maturation of imDCs. Suen et al. showed that IL-10 secreted by imDCs enhanced ESC migration and promoted angiogenesis by secreting proangiogenic factors, leading to endometriotic lesion growth (98). Izumi et al. analyzed cell surface markers of peritoneal DCs from patients with endometriosis and developed a co-incubation system of myeloid DC (MDC) with dead endometrial stromal cells (dESCs) (96). They observed that the proportion of mannose receptor (MR)^+^ MDC type 1 in patients with endometriosis was increased, and the expression of IL-1β and IL-6 was elevated in the co-incubation system, indicating that MR^+^ DCs enhanced the phagocytotic ability of dESCs and stimulated inflammation, contributing to the pathogenesis of endometriosis (96).

#### 2.1.4 Neutrophils

Previous studies have demonstrated that the concentration of neutrophils in the PF of patients with endometriosis is enhanced (99). Neutrophils are recruited into the peritoneal cavity by a variety of chemokines. The evaluated concentrations of Human neutrophil peptides 1, 2, and 3 (HNP 1-3) are correlated with the increasing neutrophils count in the PF, which is associated with the severity of the disease (99). Several reports have confirmed that neutrophils can be recruited and activated by IL-8, reacting with type I IL-8 receptors (100, 101). A prospective case-control study demonstrated that the concentration of neutrophil-activating peptide (ENA)-78, which is significantly correlated with the severity of dysmenorrhea and chronic pelvic pain, in the serum and PF of endometriosis patients is higher than that in the control group (102).

The early depletion of neutrophils by the anti-Gr-1 antibody contributed to the reduction in the number of endometriotic lesions relative to the control group in a mouse model, indicating that neutrophils play a crucial role in the initial development of endometriosis (103). Notably, cytokines secreted by neutrophils, such as CXCL10 and IL-8, would lead to peritoneal immuno-inflammation and the development of endometriosis (104). In addition, neutrophils play a crucial role in angiogenesis and endometriosis development by secreting VEGF (105). Neutrophil extracellular traps (NETs) are web-like chromatin structures that participate in the pathogenesis of immune-related diseases (106). A previous report revealed that the concentration of NETs in the PF of patients with endometriosis is significantly higher than that in patients in the control group (107). Correspondingly, another report showed elevated NET levels in the serum of patients with deep infiltrating endometriosis (108). Generally, further research is warranted to confirm the hypothesis that NETs may participate in the pathophysiological mechanisms of endometriosis through chronic inflammation.

Hormones also play a role in activating neutrophils in various ways. In a mouse model treated with Zearalenone (ZEA), a Fusarium-produced toxin that acts like estrogen, the inhibition of estrogen signaling activities decreased the number of neutrophils and the expression of pro-inflammatory cytokines by suppressing the NF-κB signaling pathway, which inhibits the growth of endometriosis (109). In turn, estrogen could increase the number of neutrophils and the expression of pro-inflammatory cytokines. All-trans retinoic acid (atRA), which is synthesized in ESCs under progesterone stimulation, downregulates the expression of VEGF *via* a direct repeat 1 element located in the transcription initiation site in neutrophils, while progesterone resistance reverses this effect and promotes angiogenesis (110).

#### 2.1.5 Other components

There are increased levels of pro-inflammatory cytokines, such as TNF-α, IL-1β, IL-6, and IL-17A, in the PF and ectopic lesions of patients with endometriosis, promoting the inflammation and development of endometriosis (111–115).

IL-1β upregulates angiogenesis by increasing the expression of VEGF and IL-6 (116), and promotes neurogenesis by stimulating the expression of nerve growth factor (NGF) (117) and brain-derived neurotrophic factor (BDNF) (118), playing an essential role in the pathogenesis of endometriosis. IL-1β is involved in the activation of COX2 and the enhancement of PGE2 levels, contributing to the synthesis of estradiol through the binding of steroidogenic factor 1 (SF-1) to promoters of steroidogenic genes and aromatase genes (119). The interaction between ER-β and the inflammasome machinery increases IL-1β levels and inhibits TNFα-induced apoptosis, resulting in immune escape, the survival of ESCs, and the epithelial-mesenchymal transition pathway, which plays an essential role in the pathogenesis of endometriosis (120). Furthermore, IL-6 promotes inflammation and facilitates the occurrence and development of endometriosis. According to a study by Sharpe-Timms, IL-6 increases the secretion of haptoglobin in endometriotic lesions, which adheres to macrophages, decreases phagocytic capability, and conversely increases the expression of IL-6, forming a positive feedback loop (121). A recent report demonstrated that IL-6 activates the NOTCH1 signaling pathway *via* E-proteins, promoting endometriotic lesion development (122). However, the exact mechanisms involved remain poorly understood. IL-17A triggered the expression of angiogenic factors, pro-inflammatory cytokines, and chemotactic cytokines, including granulocyte colony-stimulating factor (G-CSF), CXCL12, CXCL1, and CX3CL1, which play a crucial role in promoting the establishment and growth of endometriosis lesions (111). IL-8 plays a role in the pathogenesis of endometriosis by not only inducing the chemotaxis of neutrophils and promoting angiogenesis, but also stimulating the adhesion of endometrial cells to fibronectin, resulting in endometrial cells implants and development (123). Sakamoto Y demonstrated that treatment with gonadotrophin-releasing hormone agonists (GnRHa) could decrease the expression of IL-8 by inhibiting NF-κB signaling induced by TNF-α, which results in the suppression of inflammation and the development of endometriosis (124).

Meanwhile, the concentrations of immunosuppressive cytokines such as IL-4, IL-10, and TGF-β, which have been reported to participate in the pathogenesis of the disease through immune escape and the survival of ESCs, are also increased in the PF of patients with endometriosis (125). The enhanced expression of IL-4 stimulates the proliferation of ESCs *via* the activation of p38 mitogen-activated protein kinase (MAPK), stress-activated protein kinase (SAPK)/c-Jun kinase (JNK), and p42/44 MAPK signaling in endometriotic lesions (126). Zhang X et al. reported that the expression of IL-10 is increased in the PF of patients with endometriosis, and IL-10 gene promoter polymorphisms at -819 and -592 sites are associated with the risk of endometriosis (127). The level of TGF-β, which inhibits macrophage activation, downregulates the production of pro-inflammatory cytokines, stimulates angiogenesis by elevated VEGF levels, and promotes integrin-mediated adhesion and fibrosis *via* the Smad signaling pathway, is enhanced in the PF and endometriotic lesions of patients with endometriosis (125).

High-mobility group box (HMGB)-1, a type of damage-associated molecular patterns (DAMPs), mediates the inflammatory response by interacting with toll-like receptor (TLR)-2 and TLR-4. Yun BH et al. demonstrated that HMGB-1 inhibitors suppress the proliferation of endometrial stromal cells. Hence, HMGB-1 may play an essential role in the pathogenesis of endometriosis by regulating inflammation (128). Huang et al. reported that HMGB-1 upregulates levels of inflammatory cytokines and autophagy-related proteins (including beclin-1 and autophagy-related (ATG)-13) in endometrial lesions, contributing to endometriosis (129).

### 2.2 The adaptive immune system of endometriosis

The adaptive immune system, which is composed of T lymphocyte-mediated cellular immunity and B lymphocyte-mediated humoral Immunity, has been proven to be involved in the pathogenesis of endometriosis.

#### 2.2.1 T lymphocytes

Several reports have demonstrated that regulatory T (Treg) cells and T helper 17 (Th17) cells are critical for immune defense and immune homeostasis, which play essential roles in endometriosis (130, 131). Khan et al. observed increased numbers of Treg cells in the PF of women with advanced endometriosis and decreased percentages of Th17 cells in the PF of women with early and advanced endometriosis *via* flow cytometry (130). A previous report suggested that Th2 cytokines levels increased in the PF of patients with endometriosis, stimulated the generation of Treg cells, and inhibited the generation of Th17 cells, displaying immunosuppressive properties (131).

According to Gogacz, the percentage of Th17 cells in the PF of endometriotic patients with severe (stage III/IV) endometriosis was higher than that in patients with early-stage (stage I/II) endometriosis, which confirmed that the proportion of Th17 cells is related to the severity of endometriosis (132). The upregulation of CCL20 secretion by pro-inflammatory cytokines contributes to the migration of Th17 cells to ectopic endometrial lesions (133). Chang et al. proved that IL-27, secreted by macrophages and ESCs, induces Th17 cells to produce IL-10 *via* a c-Maf/RORγt/Blimp-1 signal, promoting the development of endometriosis (134). IL-17 produced by Th17 cells could stimulate the expression of angiogenic factors and pro-inflammatory cytokines, accelerating the progression of endometriosis (111). A recent study demonstrated that the over-expression of LncRNA H19 suppressed Th17 cell differentiation by downregulating the miR-342-3p/IER3 pathway, inhibiting ESCs proliferation and relieving endometriosis (135).

Treg cells, which are specialized in immune regulation and suppression, play a crucial role in exaggerating the development of endometriosis. A host of studies confirmed that the numbers of CD4^+^CD25^+^ forkhead box protein 3(FOXP3)^+^ Treg cells significantly increased in the peritoneal lesions of women with ovarian endometrioma compared with women without endometriosis (136, 137). On the contrary, Gogacz argued that the percentage of Treg cells did not differ significantly between patients with endometriosis and those in the control group (138). Another study demonstrated that CCL20 also stimulates the migration of CD4^+^CD25^high^FOXP3^+^ Treg cells, accelerating the progression of endometriosis (139). Tanaka conducted a Treg cell-depleted mice model by Foxp^3tm3Ayr/J^ (Foxp3^DTR^), which suggested that depleted Treg cells stimulate inflammation and angiogenesis, and accelerate the growth and implantation of endometrial lesions (140). Treg cells stimulate an anti-inflammatory environment by suppressing the immune response against ESC implantation, promoting the establishment and proliferation of ectopic lesions (141).

Furthermore, some reports demonstrated that cytotoxic T lymphocytes participate in the pathogenesis of endometriosis. The reduced cytotoxicity of cytotoxic T lymphocytes contributes to the immune escape of endometriotic cells. In addition, the Fas ligand (FasL) expressed by endometriotic cells binds to Fas expressed by cytotoxic T lymphocytes, inducing apoptosis of cytotoxic T lymphocytes (142).

Hormonal alterations may play a role in the suppression of activated T cell subpopulations and the impairment of NK cytotoxicity. Wu MY et al. observed that the concentrations of activated T cell subpopulations, including CD3^+^CD69^+^ T cells and CD3^+^CD25^+^ T cells, increased in the PF of patients with endometriosis after long-term GnRHa treatment, resulting in NK activity restoration (143).

#### 2.2.2 B lymphocytes

Numerous studies have reported that the number of B cells increased in the PF of patients with endometriosis (144, 145). In contrast, some reports demonstrated that there is no significant difference in the B cells count of PF between endometriosis patients and patients in the control group (146). A few other studies have also demonstrated decreased numbers of B cells (147). A study conducted on a mouse model proved that B cells depletion using Ibrutinib inhibited the activity and growth of endometriotic lesions (148). So far, little is known about the mechanism by which B cells participate in the development of endometriosis.

Previous studies suggested that estrogen could stimulate antibody production by B cells, promoting the development of autoimmune diseases (149). Similarly, some experts supported the hypothesis that anti-endometrial antibodies produced by B cells contribute to chronic inflammation and accelerate the progression of endometriosis. A previous study revealed that the number of B-1 cells increased in the PF of endometriosis patients with antinuclear antibodies (ANAs) compared to patients without ANAs and individuals in the control group (who did not have endometriosis). It demonstrated that autoantibodies produced by B-1 cells might play a crucial role in the pathogenesis of endometriosis (150). Wild et al. observed the presence of antibodies in the endometrium by indirect immunofluorescence for the first time, and revealed that anti-endometrial antibodies bind to the glandular component of ectopic and eutopic endometrium (151, 152). While most reports demonstrated increased anti-endometrial antibody titers in serum, only a few reports demonstrated these increased titers in the PF. A previous study demonstrated that the concentrations of IgG and IgA increased in the PF of patients with endometriosis compared to those in the control group; however, the differences were not statistically significant (153). Some reports supported that the serum levels of anti-endometrial antibodies are correlated with the severity of endometriosis; thus, these antibodies could be used as blood biomarkers for the diagnosis of endometriosis (154). Additionally, considering that anti-endometrial antibodies are associated with infertility (155), further studies need to be conducted to investigate whether anti-endometrial antibodies contribute to endometriosis-related infertility and identify the mechanism by which they contribute to it (if indeed they contribute).

On the other hand, cytokines secreted by B cells (such as IL-6, IL-17, and IFN-γ) also participate in the pathogenesis of endometriosis (111, 156). Additionally, the overexpression of B cell lymphoma 6 (BCL6) in the PF has been proven to promote the development of endometriosis and to be associated with endometriosis-related infertility (157). Another previous study demonstrated that Sirtuin 1 (SIRT1) and BCL6 overexpressed and suppressed the promoter of GLI1, contributing to progesterone resistance and the pathogenesis of endometriosis (158).

## 3 The effect of regulating the immune microenvironment of endometriosis

From the above, it can be seen that the peritoneal immune environment plays a central role in the pathogenesis of endometriosis. The gold standard technique for the diagnosis of endometriosis remains laparoscopic visualization with histologic verification, and the main treatment methods are still surgery and hormonal therapy (1). There is an average initial diagnostic delay of 8-10 years, which worsens the prognosis, increases the risk of recurrence, and reduces the life quality of patients. New diagnostic biomarkers and therapeutic targets are urgently needed to improve the early diagnosis and treatment of endometriosis. Therefore, it is beneficial to regard immune mediators as diagnostic tools and regulate the immune environment to treat patients with endometriosis.

### 3.1 Biomarkers for noninvasive diagnosis

There are a variety of potential biomarkers for the noninvasive diagnosis of endometriosis, including cytokines, inflammatory mediators, transcriptomics, genomics, proteomics, and metabolomics. However, none of them has been proven to be a sensitive and reliable diagnostic biomarker.

Cytokines and inflammatory mediators such as IL-1, IL-6, IL-8, IL-12, IFN-γ, MCP-1, TNF-α, sTNFR-1, and CCL5 (RANTES), which are measured in the serum and PF of patients with endometriosis, have the potential to serve as diagnostic biomarkers in patients with endometriosis (23, 159). Considering that the elevations in the levels of these mediators can be seen in multiple inflammatory diseases aside from endometriosis, the models of combined tests are more accurate and specific than those of a single test. Due to the increased concentrations of glycodelin-A in serum and PF, higher levels of IL-6 and IL-8 in the PF, and lower levels of leptin in the PF of patients with endometriosis, Kocbek V et al. proposed a few diagnostic models (160). For instance, the leptin/glycodelin-A ratio and ficolin 2/glycodelin-A ratio in serum, the biglycan/leptin ratio and RANTES/IL-6 ratio, and the ficolin2/glycodelin-A ratio accompanied by the concentration of IL-8 (160).

Serum cancer antigen (CA)-125 can be a preoperative predictor of extensive endometriosis lesions, like severe adhesions and ruptured endometriotic cysts. The sensitivity and specificity of the diagnosis can be improved through combined testing with other molecules (159). Moreover, it is reported that the neutrophil-to-lymphocyte ratio (NLR) adjuvant to serum CA-125 is regarded as a potential diagnostic tool correlated with the severity of endometriosis (161). In contrast, Kim argued that the NLR and serum CA-125 levels were not associated with the severity of endometriosis (162). The combined test of CCR1 mRNA, MCP-1, and CA-125 in serum has been proven efficient for the diagnosis or exclusion of endometriosis, with 92.2% sensitivity and 81.6% specificity (163).

Macrophages play essential roles in the etiology of endometriosis. A previous study revealed that five genes associated with M2 macrophages have a potential diagnostic value with the help of the CIBERSORT and WGCNA algorithms. For instance, the expression of bridging integrator 2 (BIN2), CCR5, and macrophage mannose receptor1 (MRC1) increased, and the expression of spleen tyrosine kinase (SYK) and metalloproteinase 12 (ADAM12) decreased in endometriosis tissues compared to non-endometriosis tissues (164). However, more studies are warranted to identify these five genes that could be used as diagnostic biomarkers in clinical settings. A case-control study demonstrated that the macrophage migration inhibitory factor (MIF) protein level was significantly higher in patients with endometriosis compared to those in the control group, and this biomarker was associated with the severity of endometriosis. According to this study, MIF may be a potential serum biomarker to be used in the diagnosis of endometriosis and the detection of its severity (165). Furthermore, the concentration of VEGF-A, a kind of angiogenic growth factor secreted by macrophages and other cells, decreased after the surgical excision of endometriosis lesions. Mohamed ML et al. proved that the measurement of VEGF-A titers in peripheral blood is more effective than the measurement of CA-125 titers in diagnosing advanced endometriosis (at a cut-off of 680 pg/ml) after surgery (166).

A recent study proposed that active-screen plasma nitriding (ASPN) can be used as a diagnostic biomarker and a potential therapeutic target in endometriosis, which is negatively correlated with the percentage of cytotoxic lymphocytes and NK cells and positively correlated with the percentage of T cells, B cells, fibroblasts, and endothelial cells by the Least Absolute Shrinkage and Selection Operator (LASSO) regression and generalized linear models (GLMs) from the GSE141549 datasets (167). Notably, TLR-2, which is essential in the innate immune system, was upregulated in the myeloid dendritic cells and B cells in patients with endometriosis. TLR-2 has been proposed as a potential noninvasive biomarker (168).

### 3.2 Immunomodulator treatment

There are many immunotherapy targeting macrophages to regulate the peritoneal immune microenvironment in endometriosis. Since macrophage depletion by intraperitoneal injection of liposomal alendronate inhibited the growth of endometriosis lesions, macrophage depletion in humans might be a potential treatment for endometriosis patients (169). CD47-SIRP-α) is considered a macrophage-associated immune checkpoint, and the expression of CD47 is increased in the peritoneal cavities of patients with endometriosis. Li et al. demonstrated that targeting CD47 could increase the phagocytic capacity of macrophages by blocking the CD47-SIRP-α pathway, which inhibits the development of endometriosis (170). Besides, Cui et al. identified 23 small molecules related to macrophages that promote endometriosis progression as potential treatment targets from the cMAP database. The top three small molecules, Celastrol, MS-275, and dexverapami, which have high absolute enrichment values, are selected as potential therapeutic agents. With anti-tumor and anti-inflammatory activity, these target molecules could be helpful in the management of tumors and endometriosis (164).

Given that ROS produced by macrophages promote endometriosis, antioxidants may be used to suppress oxidative stress and endometriosis (171). Vitamin C, a water-soluble antioxidant, has been proven to reduce endometriotic cyst volume and weight in mouse models (172). Some human clinical studies have demonstrated that vitamins C and E inhibit oxidative stress and decrease chronic pelvic pain and dysmenorrhea in patients with endometriosis (173, 174). Curcumin, an antioxidant and anti-inflammatory agent, can reduce the expression of ROS and NO to suppress oxidative stress and inhibit the NF-κB pathway and the expression of TNF-α, IL-1, IL-6, and IL-8 in macrophages of mouse models; thus, it has a potential therapeutic effect on endometriosis (175). Other antioxidants like Melatonin and N-acetyl-l-cysteine (NAC) also positively suppress oxidative stress and relieve endometriosis-related pain (176–178).

VEGF, which is produced by macrophages and other immune cells promotes angiogenesis and induces endometrial tissue attachment and implantation. Resveratrol has been proven to suppress the expression of VEGF in the PF and reduce endometriotic lesion volume in animal models, both *in vivo* and *in vitro* (179). Laschke MW reported that Epigallocatechin-3-gallate (EGCG), the major component of green tea, inhibited the development of endometriotic lesions by suppressing the proliferation and VEGF expression of endometrial cells as well as angiogenesis and blood perfusion (180). Liu Y et al. established a mouse model and found that Crocin inhibited inflammation, angiogenesis, and the growth of endometriosis lesions through decreased expression of VEGF, proliferating cell nuclear antigen (PCNA), and inflammatory cytokines in the lesions (181).

Considering that NK cells participated in the pathogenesis of endometriosis due to decreased toxicity, the activation of these cells could be a strategy to treat endometriosis. An experiment involving the intraperitoneal injection of *Lactobacillus gasseri* OLL2809 (a probiotic lactobacillus that stimulates IL-12 production) for 21 days resulted in the activation of NK cells toxicity and a reduction in the number of ectopic endometriotic lesions in mouse models. They also conducted a randomized, double-blind, placebo-controlled study and showed that L. gasseri OLL2809 could relieve endometriosis-associated pain (182). On the other hand, due to the upregulated expression of the inhibitory receptors observed in endometriosis, the inhibition of KIR2DL1, LILRB1/2, and NKG2A by monoclonal antibodies may have therapeutic effects on endometriosis (183). Moreover, the reduction of TGF-β levels could be another potential treatment option (183). Besides, stimulating NK cells with intraperitoneal injections of IL-2 has been proven to decrease endometriosis lesion sizes in the rat model (184). Although there is no available evidence in humans, it is a potential treatment option for endometriosis. Notably, the Bacillus Calmette–Guérin (BCG) tuberculosis vaccine, which has been proven to recruit NK cells, increase cytotoxicity, and reduce ectopic endometrial lesions in animal models, has been proposed as a treatment option (185).

Considering the increased expression of ER in ESCs and macrophages, we can explore drugs that regulate ER levels. In a baboon model of endometriosis, simvastatin has been proven to inhibit the growth of endometrial lesions by downregulating the expression of ER-β and upregulating the expression of ER-α *via* the inhibition of the mevalonate pathway (186). Another study reported that oleuropein, a natural product, selectively inhibited ER-β, suppressing the growth of endometriotic lesions by inhibiting the proliferation and activating the apoptosis of ESCs and improving fertility by partly recovering the impaired decidualization in mouse models (187).

## 4 Discussion

Endometriosis, a chronic inflammatory disease characterized by the implantation of endometrium-like tissues outside the uterine cavity, affects approximately 10% of reproductive-age women. Considering that the retrograde menstruation theory could not explain the fact that not all women with retrograde menstruation have endometriosis, we discussed the immune factors involved in the peritoneal immune microenvironment of endometriosis in this review.

In healthy women without endometriosis, immune cells, such as macrophages and NK cells, are recruited and activated, after which they eliminate debris after the reflux of endometrial tissues into the peritoneal cavity. Besides, chemokines secreted by immune cells contribute to a pro-inflammatory environment by inhibiting the implantation of ectopic endometrial lesions. As a result, the abnormal peritoneal immune environment plays an essential role in the pathogenesis of endometriosis.

The innate immune system participates in the pathogenesis of endometriosis. Macrophages in the PF of patients with endometriosis have decreased phagocytotic ability and produce inflammatory cytokines and angiogenesis factors, contributing to ectopic cell survival, vascularization, and fibrogenesis of endometriotic lesions, which accelerate the implantation and development of ectopic lesions. Recent studies have shown that the interaction between macrophages and nerves promotes the pathophysiology of endometriotic events. The reduction in the numbers and cytotoxicity of NK cells in the PF of endometriosis patients caused by the increased expression of inhibitory receptors, such as NKG2A, KIR2DL1 and LILRB1/2, immune checkpoint PD-1/PD-L1, TGF-β, IL-6, and IL-10, contribute to immune escape and ectopic endometrial tissue implantation. Dendritic cells are also involved in the promotion of angiogenesis and lesion formation in endometriosis. In addition, neutrophils are recruited early and secrete VEGF and NETs; thus, they play a crucial role in the initial formation and angiogenesis of endometriosis lesions. There are other components of innate immunity, including TNF-α, IL-1β, IL-6, IL-17A, ICAM-1/LFA-1, and HMGB-1, that participate in the pathogenesis of endometriosis.

On the other hand, the adaptive immune system also plays a crucial role in the pathogenesis of endometriosis. T lymphocytes mediated cellular immunity is involved in endometriosis pathophysiology, which stimulates the generation of Treg cells while inhibiting the generation of Th17 cells, contributing to immunosuppression and ESCs proliferation and invasion. The anti-endometrial antibodies and chemokines secreted by B cells, such as IL-6, IL-17, and IFN-γ, also participate in the pathogenesis of endometriosis.

Endometriosis is an estrogen-dependent chronic inflammatory disease in whose pathogenesis endocrine-immunological interactions play an indispensable role. Estrogen overexpression and progesterone resistance result in peritoneal immune microenvironment dysfunction. The enhanced expression of ER-α and ER-β induces macrophage recruitment and M2 polarization, and decreases the phagocytic capacity and expression of pro-inflammatory cytokines, inhibiting the inflammatory response. Estrogen inhibits the cytotoxic activity of NK cells on account of low autophagy of ESCs, promoting the immune escape of ESCs and the development of endometrial lesions. Hormones also play an essential role in affecting the activity of neutrophils, T cells, and Bells. They also affect the expression of pro-inflammatory cytokines.

Hormonal therapy, including combined oral contraceptives, progestogens, antiprogestogens, GnRHa, and aromatase inhibitors, is the commonly prescribed pharmacological treatment (188). Although these drugs could lead to significant pain relief, the recurrence of pain after discontinuing the medication and side effects are essential issues for these treatments. Due to its impact on suppressing ovulation and inducing amenorrhea *via* the regulation of the endocrine system, it is unavailable for patients with fertility intentions. Furthermore, some hormone medications, such as progestogens and GnRHa, would produce side effects, such as cardiovascular risks, amenorrhea, hot flashes, bone mineral density decline, and vulvovaginal atrophy, all of which negatively affect the patient’s quality of life (188). Immunotherapy, which could improve the peritoneal immune microenvironment and inhibit the development of endometriosis lesions, works in synergy with hormonal therapy to fundamentally relieve troubling symptoms of patients with fewer endocrine effects. To ensure treatment efficiency and decrease side effects, we provide future perspectives for potential diagnostic biomarkers and nonhormonal therapy based on the regulation of the immune microenvironment.

Further studies are urgently needed to comprehensively understand the immune pathophysiological mechanisms of endometriosis and explore the available diagnostic biomarkers and optimal immunological therapeutic strategies targeting endometriosis to alleviate pain, improve fertility, and enhance patients’ quality of life.

## Author contributions

SC searched the literature, generated the figures, and prepared the manuscript. YuL searched the literature and revised this manuscript. ZZ, CW, YL participated in the design of this research. XZ initiated and supervised the study and edited the manuscript. All authors contributed to the article and approved the submitted version.
